# From an indirect response pharmacodynamic model towards a secondary signal model of dose-response relationship between exercise training and physical performance

**DOI:** 10.1038/srep40422

**Published:** 2017-01-11

**Authors:** Thierry Busso

**Affiliations:** 1 Univ Lyon, UJM-Saint-Etienne, Laboratoire Interuniversitaire de Biologie de la Motricité, EA 7424, F-42023, Saint-Etienne, France

## Abstract

The aim of this study was to test the suitability of using indirect responses for modeling the effects of physical training on performance. We formulated four different models assuming that increase in performance results of the transformation of a signal secondary to the primary stimulus which is the training dose. The models were designed to be used with experimental data with daily training amounts ascribed to input and performance measured at several dates ascribed to output. The models were tested using data obtained from six subjects who trained on a cycle ergometer over a 15-week period. The data fit for each subject was good for all of the models. Goodness-of-fit and consistency of parameter estimates favored the model that took into account the inhibition of production of training effect. This model produced an inverted-U shape graphic when plotting daily training dose against performance because of the effect of one training session on the cumulated effects of previous sessions. In conclusion, using secondary signal-dependent response provided a framework helpful for modeling training effect which could enhance the quantitative methods used to analyze how best to dose physical activity for athletic performance or healthy living.

Mathematical models of athletic training and performance exist to analyze and thus optimize physical training programs^1^,^2^,^3^,^4^. They were designed as a method of studying the dynamics of changes in physical performance over time as a function of training as the dose-response effect of training is important not only for athletes but also when designing training programs aimed at improved health or fitness.

The most widely used model considers that the performance response to a work session is the combined results of the negative (fatigue) and positive (improved fitness) effects of the training session^1^, both components being modeled in an identical fashion using first-order kinetics. During training, each component increases as a function of their respective gain and then decreases at a rate that is a function of their respective time constants. Performance is assumed to be the balance between these negative and positive components. A decrease in performance will occur immediately after a session if the increase in fatigue is greater than the body’s adaptation to the workload. However, when the negative effects of fatigue are less than adaptation then the body will not only recover its initial performance level but performance will be enhanced. The impulse response is thus characterized by a rebound effect after an initial decrease in performance. Goodness-of-fit analysis showed that the original model proposed by Banister *et al*.^1^ allowed good description of the dynamics of changes in performance with training for a wide range of sports including running^5^, swimming^6^,^7^,^8^,^9^, triathlon^10^, weightlifting^11^ and hammer throwing^12^. Theory and data from the model can be used to predict the response to training, allowing the design of optimal training programs for athletes just before key competitions^13^,^14^. The model has also been used to design a rehabilitation program for a patient with coronary artery disease^15^.

Modifications to the original model were proposed to take into account a diminution in the effectiveness of training when training amounts increased^16^,^17^. This assumed a variable dose-response with the negative effects of a training session varying as a function of an accumulation of training^2^. This model considered that after repeated training sessions, the body’s capacity to benefit from a single session were impaired with it being necessary to reduce the amount of training to allow the athlete to recuperate his or her tolerance to exercise and so respond more effectively to each session. Intense training can attenuate and/or delay the rebound effect after a session but this phenomenon can be reversed if training amounts are reduced and training can again be better assimilated by the athlete. It is crucial to use this variable dose-response effect to adapt training loads to an athlete’s ability to cope with such a load and so optimize his or her training program. As this model takes into account the capacity to adapt to a new training session, a better fit of performance is observed than with the original model showing its usefulness for predicting responses to training with varied regimens^2^. The variable dose-response allows extensive analysis of the factors influencing the optimal characteristics of training before competitions^18^ and has been used to study responses to training in athletes^19^,^20^,^21^.

In the field of pharmacodynamics, indirect response models have been proposed based on the turnover of the physiological effects of a drug^22^,^23^. Such turnover models were developed to describe rebound phenomena and the development of tolerance^24^,^25^. Drug tolerance is defined as attenuation of a response to a given dose due to prior exposure. Indirect response models take into account the processes that inhibit or stimulate the factors controlling the response or resulting in tolerance or rebound phenomena. Administration of a drug is assumed to provoke changes in response depending on the amount of a precursor which may have accumulated or been depleted as a function of past administration of the drug. It has been shown that the response patterns obtained with precursor-dependent indirect response models are useful for describing changes to the response profile of a drug^24^,^25^.

There is a clear analogy between the biological response to repeated drug exposure and changes in performance with repeated training sessions. Adaptation to training is defined as changes in structure and function resulting from repeated bouts of exercise which prepare the body to better cope with exercise^26^. A training session leads to cellular disruption which, during post-exercise recovery, activates the multiple signaling pathways involved in the phenotypic plasticity specific to the mode of exercise^27^. We can consider these as secondary to the primary stimulus of the exercise as these signals continue to drive training-induced adaptations after cessation of the exercise. Similarly to a precursor of the biological effect of a drug, it is this secondary signal which is the agent that translates the primary training stimulus into training-induced adaptation as depicted in Fig. 1. Acute exposure to exercise is the primary stimulus for training-induced adaptations that in turn activate a secondary signal which then dissipates during post exercise recovery. The cumulated signal resulting from repeated exposure to exercise increases the production of the training effect counterbalancing the loss of adaptations. Performance will improve when the amount of training produces effects at a greater rate than their removal and conversely, when these effects are removed faster than they are produced, then reduced performance may be observed.

The secondary signal model assumes that the gain in performance after a single training session peaks several days after the exercise as the secondary signal continues to stimulate adaptations within the body. Although the assumed variation in performance after one training session is similar to that described by the model with two antagonistic components^1^, there is a difference in that the gains between the positive and negative functions of this model give a decrease in performance for a few days immediately after the training session due to the acute fatigue induced by the exercise. Since previous models have evidenced the importance of cumulated fatigue with training, the model described in Fig. 1 ought also to introduce the negative effect of training on performance. The Banister *et al*.^1^ and variable dose-response^2^ models considered the negative effect of training to be fatigue which counterbalanced the positive effect of the exercise. The secondary signal model gives us the opportunity to test an alternative explanation. Although acute fatigue occurs for only a few days after a training session, the various models propose that the time required for athletes to recover performance levels is in the range of several weeks^6^,^7^,^8^,^9^,^12^. A time frame of several weeks corresponds instead to the amount of time needed to recover from overreaching or overtraining, which refer to the decrease in performance resulting from maladaptation to a period of excessive training with inadequate recovery^28^,^29^,^30^. The use of secondary signal-dependent responses allows us to formulate models that can distinguish between acute fatigue counterbalancing performance and maladaptation to excessive training loads. The latter can be added to the model assuming that cumulated training diminishes the positive effect produced by a given signal through an inhibition process.

We hypothesized that using secondary signal models would enhance the quantitative methods used to analyze how to dose levels of physical activity for athletic performance or healthy living and so designed several secondary signal models for modeling the effects of physical training and comparing their ability to describe the dynamics of responses to varying training regimens. The models used in this study differ in their description of the negative effects of training; they consider both fatigue as the counterbalance to the positive effects of training on performance and the inhibition of training-induced adaptations that is responsible for maladaptation to intensified training. These models were then tested using data from an earlier report^2^.

## Secondary signal models

### Formulation of models

**Figure 2:**
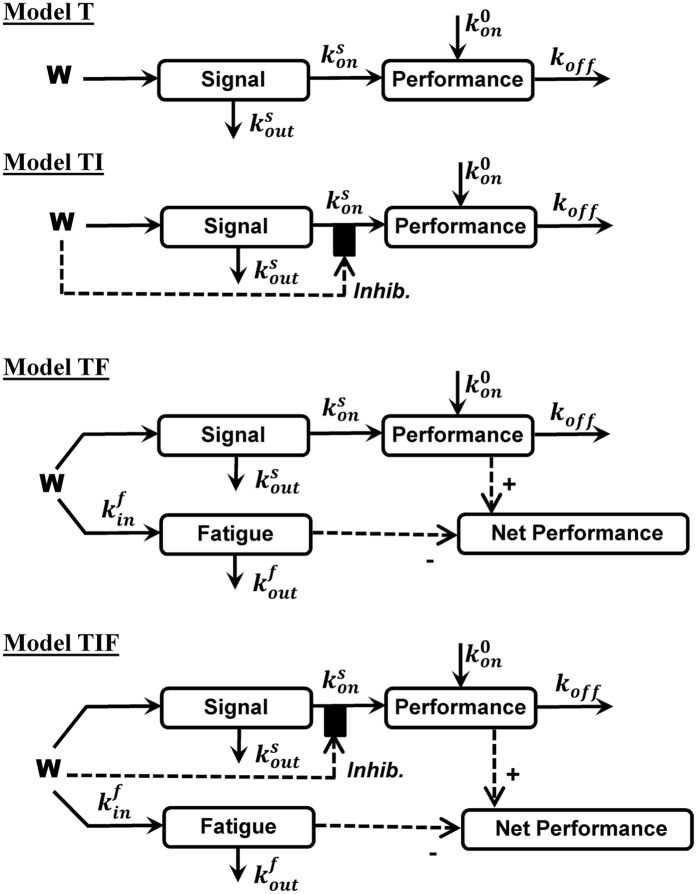
Secondary signal models tested in this study: Model T with signal-dependent production of performance, Model TI adding to Model T inhibition process that reduces production of performance, Model TF adding to Model T fatigue process that reduces net performance with time and Model TIF adding to Model T both inhibition and fatigue processes.

The first step was to build models assuming that change in performance results from training effect (*i.e.* production of performance) counterbalancing loss of adaptation (*i.e.* removal of performance). They are all based on an indirect response to the primary training stimulus, as it is the secondary signals that stimulate the training effect. Additionally, training could also act negatively by inhibiting these secondary signals that drive the training effect or because fatigue counterbalances the positive effect of the exercise. To test these different hypotheses, four models were formulated and compared (Fig. 2).

The basic scheme of the proposed models is that the effect of training on performance (*Perf*) is the sum of the cumulated responses to each training bout produced by an indirect mechanism such as the stimulation or inhibition of the production of an effect counterbalanced by its dissipation. *Perf* is the response to training ascribed to a performance criterion measured frequently throughout the period under study.

The change in performance over time with no training can be described as


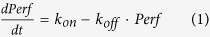


where *k*_*on*_ represents the zero-order rate variable for production of performance and *k*_*off*_ the first-order rate constant for loss of performance.

Stimulation of the production of performance (*Prod*) occurs dependent on the amount of training (*W*) quantified from the duration and intensity of the exercise done during each training session with production of a secondary signal (*Signal*) equal to the amount of training. The signal then dissipates with a first-order rate constant 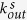. The secondary signal is transformed into performance with a first-order rate constant 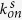, adding to the baseline value 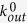. As a result, the rate of change in *Signal* after one training session, and before the next one, is given by


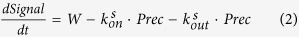


At any time, the production of performance is


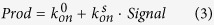


Model T represents the simplest process where the responses to training are described by the production of the secondary signal which is the mediator for the change in performance through production counterbalancing its removal.

Model TI adds to Model T a process that inhibits production of performance by the secondary signal according to the function *Inhib* introduced in equation (3) which becomes


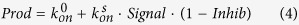


Inhibition on a given day is proportional to the amount of training done on this day. It therefore follows that


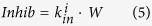


where 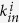 is the constant of proportionality.

Model TF adds to Model T a process of fatigue counterbalancing the positive effect of training. The net performance (performance minus fatigue) represents the observed response to training.

During a training session, the amount of training leads to a proportional production in fatigue (*Fatigue*) at the rate 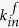 which dissipates with a first-order rate constant 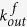. The result is


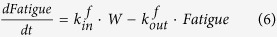


Model TIF adds to Model T both processes for inhibition of the factor controlling the production of performance and fatigue using the same assumptions as models TI and TF respectively. Its formulation thus includes equations (4) and (5) for inhibition and equation (6) for fatigue.

### Discretization of model equations

The secondary signal models of the training effects are defined above by a set of differential equations. The data required to solve them are obtained from the quantity of training performed daily by subjects over several weeks or months during which performance is measured on several different occasions. For solving the proposed models, w(t) was considered as a discrete function; *i.e.*, a series of impulses each day, *W*_*i*_ on day i, and the model performance 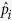 on day i was estimated by mathematical recursion from the series of W before day i. For this purpose, we discretized the continuous integral of the differential equations of each tested model as recursive sequences in which each term on a given day was defined as a function of other terms on either the same or the preceding day.

The performance on day i, *Perf*_*i*_, is computed from its level on day n − 1 and the balance between removal and production on day n − 1 as follows


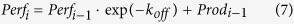


The initial value of performance *Perf*_0_ was assumed to be equal to the first estimate of performance and to be stationary and thus the baseline production, 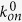, is equal to the initial rate of removal as follows


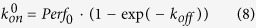


and


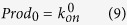


In Model T, the signal on day i, *Signal*_*i*_, is computed from its level on day i − 1 and *W*_*i*_ as follows


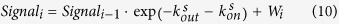


giving


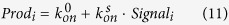


In Model TI, the term *Inhib* was added as a variable function of training amounts which diminish the production of performance. Its value on day i is computed as follows


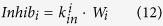


Equations 11 and 12 are modified in Model TI


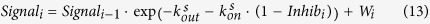


and


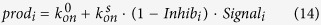


In Models TF and TIF, the equations for *Signal*_*i*_, *Prod*_*i*_ and *Perf*_*i*_ are identical to those in Models T and TI respectively. In both of them, an equation is added to the term ascribed to fatigue. Its level on day i, *Fatigue*_*i*_, is computed as follows


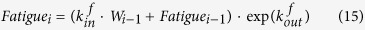


with *Fatigue*_0_ and *W*_0_ initialized to 0.

In both Models TF and TIF, model output is net performance as follows


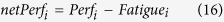


## Results

Table 1 gives the indicators of goodness-of-fit for the four models tested in this study which were statistically significant for each subject (P < 0.001). F-tests on residual variance showed that Model TI improved the fit in all subjects (P < 0.001) and Model TF only in subject 3 (P < 0.001) in comparison to model T. Model TIF improved the fit in 4 subjects (P < 0.05 in subjects 2, 4 and 6 and P < 0.001 in subject 3) in comparison to model TI.

AICc for Models TI and TIF was lower than for Models T and TF in 5 subjects. AICc was the lowest for Model TF only in subject 3. Average weight of evidence calculated from w(AICc) was close for models TI and TIF (0.428 ± 0.397 and 0.479 ± 0.302 respectively), whereas weak weights were found for Models T and TF (0.000005 ± 0.000013 and 0.092 ± 0.226 respectively). Nevertheless, the estimates for 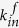 with Models TF and TIF were positive only in subject 3. Obtaining negative value of 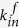 in the remaining five subjects was not consistent with the hypothesis that the fatigue term counteracts the positive effect of training.

**Figure 3:**
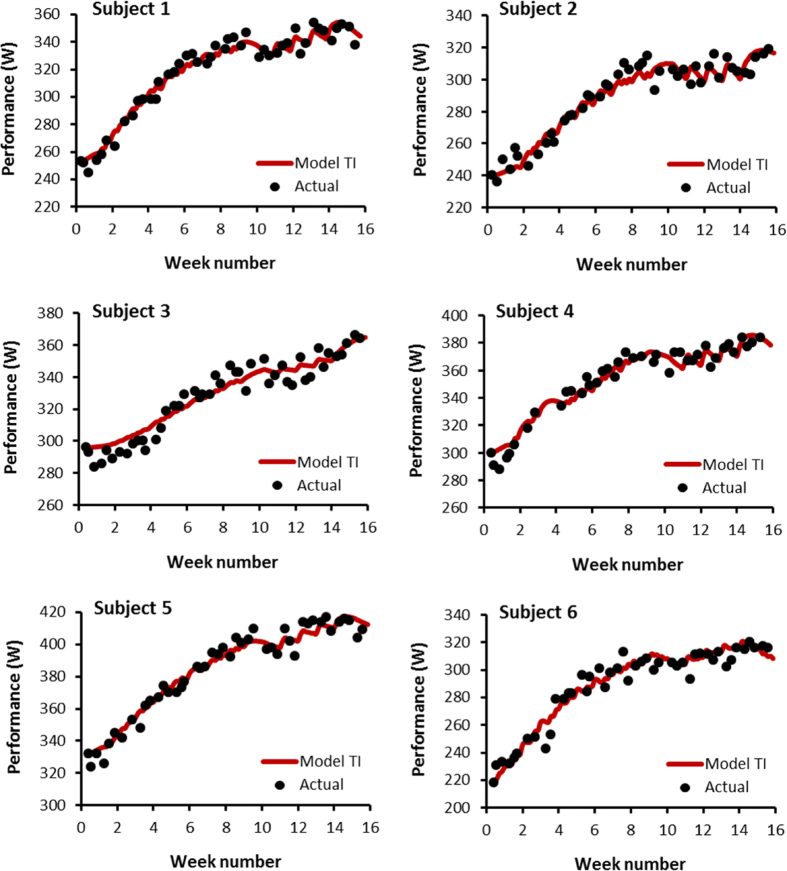
Measured performance around line of best fit with Model TI for the 6 subjects.

**Figure 4:**
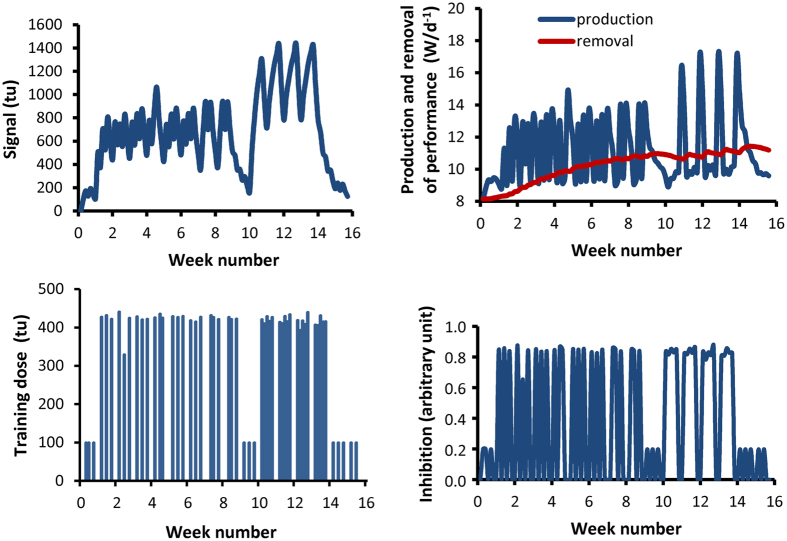
Results for Model TI in subject 1. A: measured performance around line of best fit. B: rate of production and removal of performance. C: Amount of signal producing performance. D. Inhibition variable which reduces production of performance from signal.

**Figure 5:**
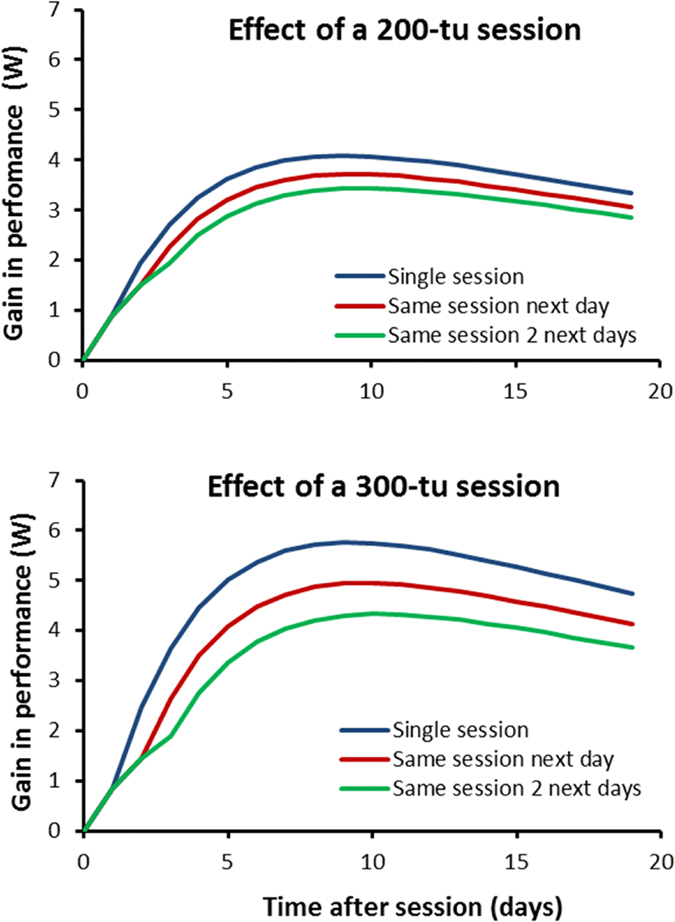
Gain in performance after a training session (200 and 300 tu): (1) no work the days after (single session), (2) same training session one day after and (3) same training session two days after. Computations were made with Model TI with the median values of parameter estimates.

**Figure 6:**
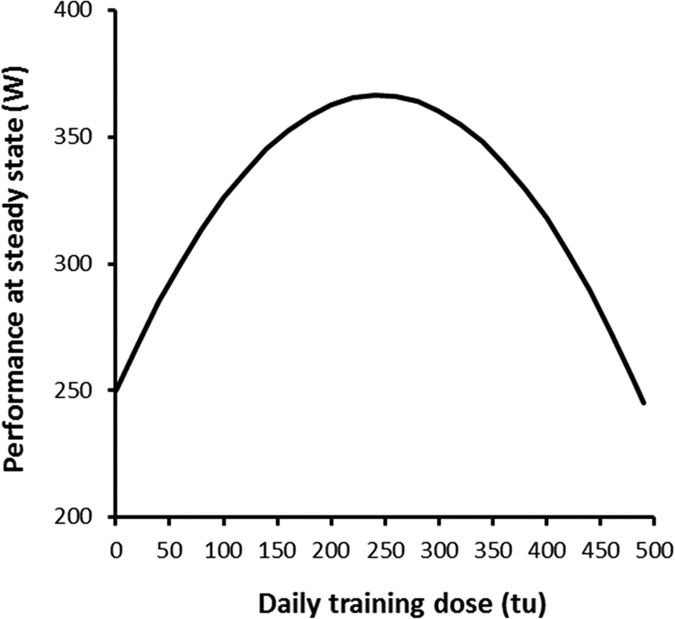
Performance at steady state for same training dose repeated each day with baseline performance equal to 250 W. Computations were made with Model TI with the median values of parameter estimates.

Since evidence for the validity of the models including the fatigue term was only found in subject 3, analysis of the statistics was in favor of opting for Model TI from the four models tested in this study. Figure 3 shows the fit of Model TI to actual performance in the 6 subjects. The data of subject 1 were selected to show the balance between production and removal of performance giving a regular increase in performance during the first period of training which was interrupted during the 5 consecutive days of training because inhibition remained at a high level (Fig. 4). Table 2 gives the estimated parameters for Model TI showing large inter-individual variations for 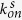 and 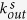 because of outliers. Median values of *k*_*off*_ 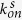, 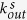 and 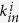 were 0.0305 (IQR 0.0055), 0.0074 (IQR 0.0031), 0.292 (IQR 0.165) and 0.0021 (IQR 0.0001) respectively. We chose the median value of the estimates to simulate the response to a 200 and 300-tu session for three cases: a single session and the same session performed on 1 and 2 subsequent days (Fig. 5). These simulations illustrate how a session alters the response to previous ones to an extent dependent on the repeated dose. As a consequence, when identical sessions are repeated sufficiently for the subject to reach a steady state, the relationship between the daily training dose and performance has an inverted-U shape as depicted in Fig. 6.

## Discussion

The goal of this study was to verify the ability of a secondary signal model to fit performance changes with training. Statistical analysis shows that the models tested in this study enable us to relate the changes in performance of each subject at P < 0.001. However, the comparison of indicators of goodness-of-fit and consistency of parameter estimates indicates that Model TI is the most useful of the four models tested.

The original model proposed by Banister *et al*. and the variable dose-response model were tested with the same data using the least square method to estimate the parameters for each subject^2^. Successive linear minimization with a grid of values for decay time constants gave the total set of parameters. In the present study, the entire set of parameters was estimated using a method designed for non-linear models. The latter could not however be used to fit the two previous models because the solution was sensitive to the starting value for time constants chosen to initialize the computation. It could be the result of ill-conditioning problems revealed for the Banister *et al*. model by analysis of the correlation matrix of the parameter estimates^7^. This is why we did not include these two other models in the present study. In addition to better model conditioning, using secondary signal-dependent responses gave a goodness-of-fit as satisfactory as with previous models. By way of comparison, Adj.R^2^ for the model proposed by Banister was 0.857 ± 0.042 and for the variable-dose response model 0.944 ± 0.042 similar to those for Model T (0.883 ± 0.045) and Model TI (0.945 ± 0.019) respectively.

Model T was the simplest model tested in this study with an accumulation of the signal which depleted slowly after training session giving the training effect. Although it did not consider fatigue to act negatively on performance, Model T described performance changes with training as precisely as did the original model in which performance was calculated from two components modeled using first order kinetics. Introducing the fatigue variable balancing the effect of training in Model TF did not significantly improve the fit compared to Model T. This result implies that the secondary signal-dependent response would be a good alternative to the two antagonistic first order systems. Using secondary signal also has the advantage of avoiding the collinearity between positive and negative effects leading to ill-conditioning problems revealed with the original model from Banister *et al*.^7^.

The negative influence of training was considered in Model TI assuming that a session inhibited training-induced adaptation from the signal accumulated as a function of past sessions. This model gave a performance data fit as precise as with the variable dose-response model^2^. The latter implied that fatigue produced by a single unit of training increases when training is intensified. It results in an inverted U-shape relationship between the amount of daily training and performance gain^2^. The existence of an optimum amount of daily training was also captured by Model TI (Fig. 6). This feature was the training effect for a given signal varying as a function of the current training sequence as depicted in Fig. 5. The optimum daily training amount estimated from Model TI was however lower than the variable dose-response model (253 ± 45 vs 322 ± 70 tu per day). Adding a fatigue variable acting negatively on the training effect in Model TIF did not improve the description of performance response to training compared to Model TI. Contrary to the original concept of fatigue and adaptation balancing the performance response, the negative effect of training in Model TI acts through altering the performance gain for a training unit as a function of the work done on subsequent days. In other words, maladaptation to intensified training would be due to inhibition of the transformation of the secondary signal thus inhibiting adaptation. Because of signal dissipation, this inhibition process would be responsible for signal wasting explaining why the performance change was lower than expected with high amounts of training. However, Model TIF has the potential to single out acute fatigue after a training session and maladaptation during periods of intense training. The benefit of adding a fatigue component to the model was however not evidenced in this study, with the exception of one subject. Athletes train with workloads much higher than those by the subjects in this study. It has been acknowledged that recovery from accumulated fatigue occurring during an overload period would be a key point for taper strategies in the final stages of preparation before high-level competitions^8^,^14^,^18^,^21^. Adding acute fatigue to maladaptation to excessive training as in Model TIF would deserve to be re-examined for athletes.

In conclusion, this study shows that secondary signal-dependent response provides a useful framework to model the dynamic response of performance during training. The comparison of the different models highlights the usefulness of Model TI which considers inhibition of current training effect with new training sessions. It is worth noting that the data used in this study came from healthy volunteers for an intensified training program but Model T would also be appropriate for studying the response to lower training doses during rehabilitation programs. On the other hand, Model TIF could be more appropriate for the analysis of responses in elite athletes because of the higher workload during their preparation. Further studies are warranted to determine how secondary signal models could create new opportunities to quantify the dose-response effect of exercise in order to gain insights into the prescription of physical activity programs with athletic performance, fitness or health goals.

## Methods

### Experimental data

To test the models proposed in this study, we took the experimental data from a study entirely described in a previous report^2^. This study had been approved by the local ethics committee (Conseil Consultatif de la Protection des Personnes dans la Recherche Biomédicale de la Loire) and the methods used in accordance with relevant guidelines. Six healthy men volunteered after giving their informed written consent. The 15-wk experiment included two periods of training: an 8-wk period with 3 training sessions per week (weeks 1–8) and a 4-wk period with 5 training sessions per week (weeks 10–13) separated by one week without training (week 9). The last two weeks of the experiment were also a period without training (weeks 14–15). Throughout the experiment, the subjects performed a total of 40 to 46 trials to measure the maximal work they could develop for 5 min on a cycle ergometer (Model 829E, Monark, Stockholm, Sweden). The power output developed by the subjects was averaged over the 5-min test to estimate P_lim5′_. Performance was measured 2 or 3 times each week during the entire experiment including the week before it began (week 0), and the rest periods (weeks 9, 14 and 15). During the first training period (weeks 1–8), the subjects performed one test 3 times per week to measure P_lim5′_ and a training session consisting of 4 repetitions of 5 minutes of work interspersed with 3 minutes of active recovery. During the second training period (weeks 10–13), the subjects trained on 5 consecutive days; on days 1, 3 and 5 this was identical to the first training period and on days 2 and 4, the performed 5 repetitions of 5 minutes but no performance trial. Exercise intensity was set at 85% of the last measured P_lim5′_. The daily amount of training on day i (W_i_) was computed in arbitrary units (tu) from mean power (P_mean_) during 5-min bouts of exercise (P_lim5′_) as follows


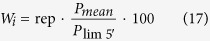


with rep the number of repetitions of the training sequence. The amount of training for each performance trial was fixed at 100 tu.

The entire data set for the 6 subjects can be found as Supplementary Table S1.

### Model fitting and statistics

Model performances 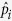 were ascribed to the effects of training for Models T and TI and to net effects for Models TF and TIF and fitted to measured performance *p*_*i*_.

With n the number of performance measurements during the study and *res*_*i*_ the residuals from the fit 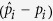. Assuming normally distributed errors, log-likelihood of the estimated model *ln(L*) was computed as follows


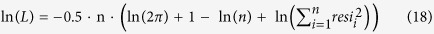


Each model was fitted for each subject by minimizing the negative log-likelihood function using nlm function in R package^31^.

For the measures of goodness-of-fit, we calculated the coefficient of determination (R^2^)


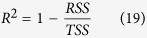


with *RSS* the residual sum of squares and *TSS* the total sum of squares. The mean square error on performance estimation (SE) was computed as RSS/(N-*p).* The statistical significance of the fit was tested by analysis of variance of the *RSS* in accordance with the degrees of freedom of each model.

The adjusted coefficient of determination (Adj . R^2^) was calculated according to p the number of parameters for each model (3 for Model T, 4 for Model TI, 5 for Model TF and 6 for Model TIF) as follows


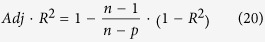


The level of confidence for each level of model complexity was tested by analysis of variance of the related decrease in residual variation. The decrease in *RSS* explained by the introduction of further model parameters was tested using the *F*-ratio test in accordance with the increase in degrees of freedom^32^.

As an indicator of goodness-of-fit, we also calculated the bias-corrected Akaike Information Criterion (*AICc*)^33^


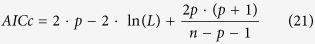


We used Akaike weights to obtain weight of evidence for each of the four models tested in this study^33^


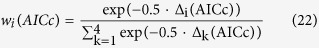


where i is the model number and Δ_i_(AICc) the difference between *AICc* of model i and the lowest *AICc.*

A spreadsheet implementation of Model T and Model TI can be found as Supplementary Information.

## Additional Information

**How to cite this article**: Busso, T. From an indirect response pharmacodynamic model towards a secondary signal model of dose-response relationship between exercise training and physical performance. *Sci. Rep.* **7**, 40422; doi: 10.1038/srep40422 (2017).

**Publisher's note:** Springer Nature remains neutral with regard to jurisdictional claims in published maps and institutional affiliations.

## Supplementary Material

Supplementary Information

Supplementary Spreadsheet

## Figures and Tables

**Figure 1:**
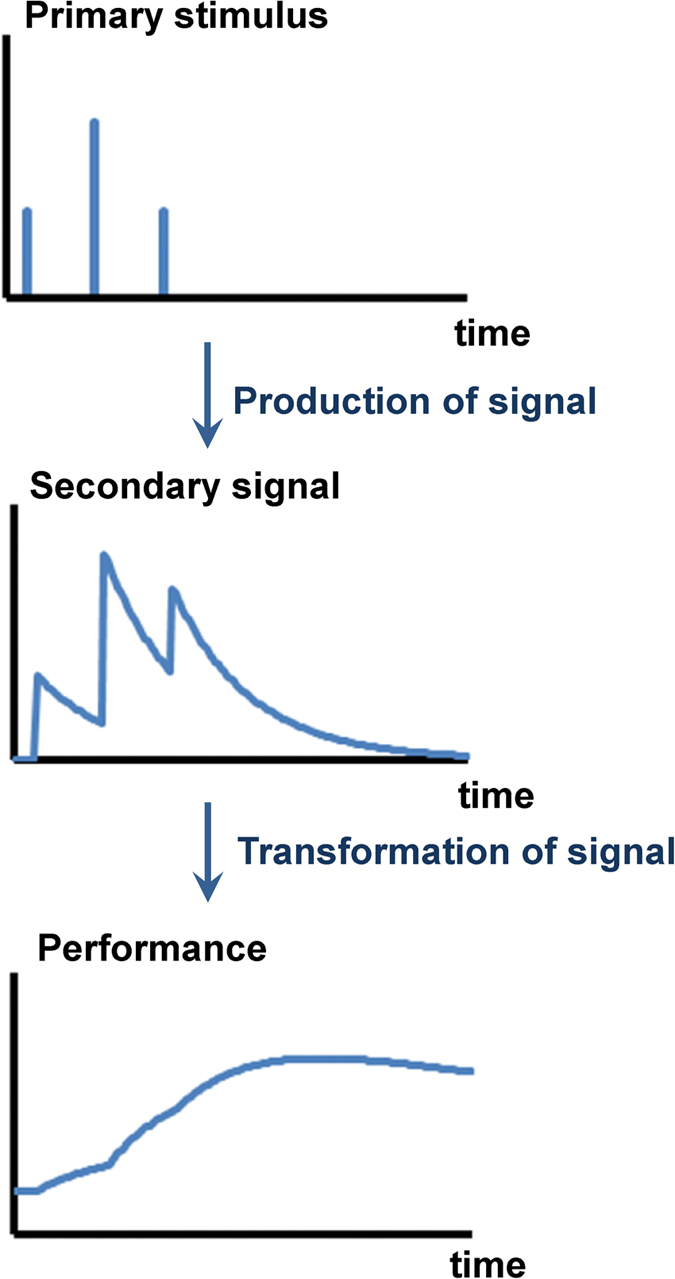
Schematic representation of secondary signal model of training effect. Impulse training doses are the primary stimulus giving a secondary signal which accumulates with training before its dissipation. The signal is transformed into training effect (*i.e.* production of performance). Performance increases because the production is greater than the loss of training-induced adaptation (*i.e.* removal of performance).

**Table 1:** Indicators of goodness-of-fit of secondary signal models.

		Model T	Model TI	Model TF	Model TIF
	**Adj.R**^**2**^	0.923	0.967	0.919	0.967
Subject 1	**AICc**	328.04	291.07	332.74	293.96
	**w(AICc)**	0.000	0.809	0.000	0.191
	**Adj.R**^**2**^	0.860	0.932	0.853	0.939
Subject 2	**AICc**	330.27	299.27	335.22	297.16
	**w(AICc)**	0.000	0.258	0.000	0.742
	**Adj.R**^**2**^	0.896	0.920	0.946	0.947
Subject 3	**AICc**	323.71	312.77	296.40	296.83
	**w(AICc)**	0.000	0.000	0.554	0.445
	**Adj.R**^**2**^	0.831	0.950	0.826	0.956
Subject 4	**AICc**	312.72	265.16	316.83	263.75
	**w(AICc)**	0.000	0.331	0.000	0.669
	**Adj.R**^**2**^	0.944	0.965	0.943	0.963
Subject 5	**AICc**	301.99	281.39	305.55	287.04
	**w(AICc)**	0.000	0.944	0.000	0.006
	**Adj.R**^**2**^	0.843	0.935	0.846	0.943
Subject 6	**AICc**	351.29	312.56	353.01	310.10
	**w(AICc)**	0.000	0.226	0.000	0.774
	**Adj.R**^**2**^	0.883 ± 0.045	0.945 ± 0.019	0.889 ± 0.053	0.952 ± 0.011
Mean ± SD	**AICc**	324.67 ± 16.79	293.70 ± 18.57	323.29 ± 20.95	291.47 ± 15.51
	**w(AICc)**	0.000 ± 0.000	0.428 ± 0.367	0.092 ± 0.226	0.479 ± 0.302

**Table 2:** Estimate of parameters for Model TI.

	*k*_*off*_	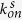	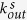	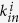
**Subject 1**	0.0328	0.00845	0.297	0.00199
**Subject 2**	0.0285	0.00462	0.189	0.00246
**Subject 3**	0.0267	0.00071	0.053	0.00148
**Subject 4**	0.0326	0.00783	0.286	0.00213
**Subject 5**	0.0194	0.00695	0.405	0.00201
**Subject 6**	0.0341	0.03751	1.066	0.00211
**Mean ± SD**	0.0290 ± 0.0055	0.0110 ± 0.0133	0.383 ± 0.355	0.00203 ± 0.0003
