# Supplementary material for: From an indirect response pharmacodynamic model towards a secondary signal model of dose-response relationship between exercise training and physical performance
Source: Sci Rep. 2017 Jan 11;7:40422. doi: 10.1038/srep40422 (PMC5225461; doi:10.1038/srep40422)
Supplement: Supplementary Information [file srep40422-s1.pdf]

Supplementary Information for:

From an indirect response pharmacodynamic model towards a secondary signal model of dose-response relationship between exercise training and physical performance

Thierry Busso

Univ Lyon, UJM-Saint-Etienne, Laboratoire Interuniversitaire de Biologie de la Motricité, EA 7424, F-42023, Saint-Etienne, France

Supplementary Table S1: Raw data used in this study: daily amount of training and performance of each subject.

Day number	Training dose Subject 1 (tu)	Performance Subject 1 (Watt)	Training dose Subject 2 (tu)	Performance Subject 2 (Watt)	Training dose Subject 3 (tu)	Performance Subject 3 (Watt)	Training dose Subject 4 (tu)	Performance Subject 4 (Watt)	Training dose Subject 5 (tu)	Performance Subject 5 (Watt)	Training dose Subject 6 (tu)	Performance Subject 6 (Watt)
1	0		0		0		0		0		0	
2	100	253	100	240	0		0		0		0	
3	100	252	0		100	296	100	300	100	332	100	218
4	0		100	236	100	293	100	291	100	324	100	231
5	100	245	0		0		0		0		0	
6	0		100	250	100	284	100	288	100	332	100	233
7	0		0		0		0		0		0	
8	427.5590551	254	0		0		0		0		0	
9	0		427.8688525	244	435.6643357	286	441.8918919	296	443.5582822	326	412.0689655	232
10	431.7829457	258	0		0		438.4615385	299	0		0	
11	0		411.2840467	257	440.1360544	294	0		431.3609467	338	437.2881356	236
12	422.3880597	268	417.4603175	252	0		438.5620915	306	0		441.4225941	239
13	0		0		446.0207612	289	0		428.115942	345	0	
14	0		0		0		0		0		0	
15	440.9090909	264	0		0		0		0		0	
16	0		425.203252	246	445.3924915	293	0		432.1637427	342	432.8	250
17	329.6703297		0		0		433.3333333	318	0		0	
18	0		320		0		333.3333333		333.3333333		328	
19	424.822695	282	0		439.7260274	292	0		0		433.0677291	251
20	0		416.2055336	253	0		440.4255319	329	426.3456091	353	0	
21	0		0		435.5704698	298	0		0		0	
22	428.6713287	286	0		0		0		0		0	
23	0		423.0769231	260	437.3333333	300	0		439.0804598	348	442.3868313	243
24	420.5387205	297	0		0		0		0		0	
25	0		415.7894737	266	433.3333333	300	0		429.281768	362	428.8537549	253
26	422.147651	298	421.8390805	261	433.3333333	294	0		0		0	
27	0		0		0		0		433.1506849	365	399.6415771	279
28	0		0		0		0		0		0	
29	426.1744966	298	0		0		0		0		0	
30	0		421.1678832	274	442.8571429	301	438.9221557	334	424.7956403	367	399.6415771	279
31	435.5704698	298	0		0		0		0		0	
32	425.4019293	311	410.4693141	277	448.0519481	308	437.2093023	344	429.4117647	374	410.9540636	283
33	0		413.6690647	278	0		0		0		415.1943463	283
34	0		0		438.5579937	319	437.3913043	345	439.4594595	370	0	
35	0		0		0		0		0		0	
36	429.1139241	316	0		0		0		0		0	

37	0		409.2198582	282	441.6149068	322	0		440.5405405	370	398.6486486	296
38	427.0440252	318	0		0		447.5218659	343	0		0	
39	0		403.4482759	290	452.7950311	322	0		441.0187668	373	416.9014085	284
40	429.6296296	324	418.3391003	289	0		438.028169	355	439.5225464	377	405.0847458	295
41	0		0		440.4255319	329	444.9856734	349	0		0	
42	0		0		0		0		0		0	
43	418.7878788	330	0		0		0		0		0	
44	0		418.3391003	289	0		446.4387464	351	0		399.0033223	301
45	415.407855	331	0		438.3685801	331	0		435.7512953	386	0	
46	0		409.7643098	297	0		438.718663	359	437.6623377	385	414.9825784	287
47	427.3846154	325	421.6216216	296	442.5076453	327	0		0		0	
48	0		0		452.5835866	329	437.9501385	361	436.7875648	386	406.0402685	298
49	0		0		0		0		0		0	
50	0		0		0		0		0		0	
51	432.0987654	324	419.4719472	303	440.4255319	329	421.0140845	355	409.8734177	395	406.9767442	301
52	427.0516717	329	0		0		433.3333333	366	0		0	
53	0		414.8387097	310	441.3489736	341	0		434.0101523	394	406.7092652	313
54	421.6617211	337	0		0		0		0		0	
55	0		420.2614379	306	438.0952381	336	430.2949062	373	431.6582915	398	423.2876712	292
56	0		0		0		0		0		0	
57	0		0		0		0		0		0	
58	427.1641791	335	0		0		430.6233062	369	425.5102041	392	410.2310231	303
59	421.6374269	342	422.0779221	308	434.2939481	347	0		0		0	
60	0		417.4193548	310	0		0		429.7029703	404	405.8823529	306
61	423.03207	343	0		439.3586006	343	429.7297297	370	0		0	
62	0		414.9206349	315	432.361516	343	0		422.1945137	401	406.4935065	308
63	0		0		0		0		0		0	
64	100	337	0		0		0		0		0	
65	0		100	293	100	331	0		100	403	100	300
66	100	347	0		0		100	366	0		0	
67	0		100	305	100	348	100	371	100	410	100	305
68	100		0		0		0		0		0	
69	0		100		100		100		100		100	
70	0		0		0		0		0		0	
71	420.9726444	329	0		0		0		0		0	
72	410.2564103		413.7254902	306	441.8803419	351	450.8379888	358	435.5163728	397	412.1311475	305
73	429.3413174	334	394.7368421		436.6812227		430.9165527		421.3836478		398.0263158	
74	417.1686747		407.2847682	302	454.7619048	336	437.8016086	373	436.6834171	398	412.8712871	303
75	427.2727273	330	394.7368421		441.6543575		423.5924933		425.5050505		406.25	
76	0		408.496732	306	434.3108504	341	437.8016086	373	445.177665	394	412.1311475	305
77	0		0		0		0		0		0	
78	414.4578313	332	0		0		0		0		0	
79	411.0612855		416.4983165	297	438.9048991	347	441.1444142	367	429.7560976	410	423.5494881	293
80	429.9703264	337	396.6942149		422.5146199		430.5177112		417.4876847		399.0066225	
81	415.6804734		415.5844156	308	439.4658754	337	444.4141689	367	432.3383085	402	407.3954984	311

82	433.9233038	339	400.990099		441.9642857		433.604336		416.3522013		385.2327448	
83	0		419.4630872	298	458.2089552	335	442.8571429	371	435.8778626	393	407.6923077	312
84	0		0		0		0		0		0	
85	418.8571429	350	0		0		0		0		0	
86	393.5389134		416.8831169	308	440.9090909	352	438.6243386	378	423.6714976	414	408.681672	311
87	417.8247734	331	384.6153846		434.7826087		429.7297297		406.2877872		394.8220065	
88	408.9552239		415.1898734	316	450.295858	338	448.0662983	362	430.2663438	413	415.3094463	307
89	439.8230088	339	388.9789303		442.4778761		429.5485636		410.6280193		390.3225806	
90	0		412.2923588	301	450.5882353	340	441.4634146	369	433.4939759	415	409.2651757	313
91	0		0		0		0		0		0	
92	407.3446328	354	0		0		0		0		0	
93	406.25		398.089172	314	444.1340782	358	439.3617021	376	417.8743961	414	416.5562914	302
94	431.4285714	350	380.0322061		427.5568182		421.192053		404.33213		397.3727422	
95	415.4727794		419.218241	307	453.7572254	346	436.6754617	379	424.2206235	417	414.0065147	307
96	416.091954	348	400.3267974		436.5192582		425.5319149		418.1818182		385.2327448	
97	0		409.5081967	305	449.2957746	355	448.5254692	373	443.1372549	408	403.7974684	316
98	0		0		0		0		0		0	
99	100	341	0		0		0		0		0	
100	0		100	304	100	353	100	384	100	414	100	315
101	100	350	0		0		0		0		0	
102	0		100	303	100	354	100	377	100	416	100	320
103	100	353	0		0		0		0		0	
104	0		100	314	100	361	100	380	100	415	100	316
105	0		0		0		0		0		0	
106	100	351	0		0		0		0		0	
107	0		100	316	100	366	100	384	100	404	100	317
108	100	338	0		0		0		0		0	
109	0		100	319	100	364	0		100	409	100	316
